# Effects of microwave or infrared radiation on protein ruminal degradation and *in vitro* intestinal digestibility in feedstuffs

**DOI:** 10.1371/journal.pone.0359231

**Published:** 2026-10-08

**Authors:** Erollykens F. Santos, Valber C. Morais, Elias A. Lopes, Marina M. Ferreira, Fernando A. A. Cidrini, Jardeson S. Pinheiro, Edenio Detmann, Marcos I. Marcondes

**Affiliations:** 1 School of Animal Sciences, Virginia Polytechnic Institute and State University, Blacksburg, Virginia, United States of America; 2 Department of Animal Sciences, Universidade Federal de Viçosa, Viçosa, MG, Brazil; 3 Department of Veterinary Medicine, Cornell University, Ithaca, New York, United States of America; 4 William H. Miner Agricultural Research Institute, Chazy, New York, United States of America; University of Georgia, UNITED STATES OF AMERICA

## Abstract

Microbial crude protein (**MCP**) is the main source of amino acids (**AA**) in the small intestine, followed by rumen undegraded protein (**RUP**). Due to limited MCP synthesis and increased AA demand in high-producing ruminants, strategies to enhance intestinal protein availability are relevant. This study evaluated the effects of thermal processing on six protein-rich feedstuffs: soybean meal, wheat bran, cottonseed meal, corn gluten feed, whole cottonseed, and whole soybean seed. A preliminary trial using soybean meal determined optimal processing times based on ruminal degradability curves. Two mathematical models (exponential and broken-line) were evaluated to determine optimal processing times for each thermal treatment. Based on the mathematical modeling performed in the preliminary trial, 30 minutes was selected for microwave treatment (**MW30**) and 92 minutes for infrared radiation (**IR92**). These time points were then applied to the remaining ingredients. These time points were then applied to the remaining ingredients. After processing, samples were incubated in situ for 16 hours in rumen-cannulated cattle, followed by in vitro intestinal digestibility (**ID**) assays. Crude protein and dry matter were analyzed, and the total protein available for absorption in the small intestine was estimated. Data were analyzed using a 6 × 3 factorial design with the GLIMMIX procedure in SAS. Whole soybean seed treated with IR92 showed the highest absorbable protein (85%), followed by whole cottonseed processed with MW30 (73.5%). Soybean meal also responded positively to both treatments, particularly IR92 (78%). Cottonseed meal showed moderate improvements, whereas corn gluten feed exhibited reductions in absorbable protein under both treatments. Wheat bran showed a negative response to MW30 but a modest improvement under IR92. Overall, the results indicate that infrared processing is more efficient for soybean-based ingredients, while microwave heating tends to be more advantageous for cotton-derived feeds, reinforcing the need to match processing conditions to ingredient-specific characteristics.

## Introduction

Livestock farming faces constant pressure to minimize environmental impacts while keeping productive and economic efficiency. One crucial aspect of this effort is reducing nitrogen (**N**) excretion, which is often associated with diets high in crude protein (**CP**) that degrade rapidly [1]. By lowering excessive N losses in feces and urine, we can improve the efficiency of protein utilization, which is one of the costliest components of animal diets [2].

Microbial crude protein (**MCP**) is considered the main source of amino acids (**AA**) in the small intestine, which may represent 50–80% of total absorbed AA in ruminants and, therefore, the main component of metabolizable protein (**MP**) [3,4]. Microbial crude protein is the result of the assimilation of N compounds from the rumen degradable protein (**RDP**) and recycled N by ruminal microorganisms. Besides having an AA profile similar to that of muscle and milk, MCP is also highly available in the small intestine [5,6]. The second main source of MP is the rumen undegraded protein (**RUP**). In general, once MCP reaches a plateau, the dietary supply of RUP may increase the flow of essential AA, consequently improving animal performance and N retention [7,8]. The limit on MCP production is caused by restrictions such as the rumen availability of energy. Therefore, the provision RUP is even more relevant for high-producing cows or growing animals, which have a greater demand for essential AA that cannot be met exclusively by MCP [6,9,10].

Many protein feeds are by-products of oilseeds (soybeans, cotton, and peanuts) after oil extraction [11]. These feedstuffs usually have between 30–50% CP; however, a large part (> 70%) is considered degradable in the rumen [12]. Thus, only a small fraction of their CP reaches the small intestine as RUP. Few products with high RUP content (>70%) are available on the market; however, their accessibility is limited due to the high costs associated with the technologies required for their production. Considering this scenario, it becomes evident that there is a need to explore viable alternatives capable of providing effective rumen protection for the protein in widely used protein-rich feeds.

The purpose of processing protein feeds is to reduce protein ruminal degradability (**RD**) without adverse effects on its intestinal digestibility (**ID**). The ID of RUP is highly variable, depending on the type of feedstuff and processing method [3,13]. One of the most studied methods to increase RUP in feeds is thermal processing, which neutralizes antinutritional factors and reduces solubility, thereby reducing fermentation and ruminal metabolism [14]. The heating causes denaturation of the protein, with consequent rearrangement of its smaller units, thus preventing access to its active sites for degradation. On the other hand, excessive heating can complex protein subunits with carbohydrates and other macromolecules through non-enzymatic reactions, making the proteins unavailable for rumen degradation and intestinal digestion [12,15].

The thermal processing must be conducted with consistent, standardized procedures, using appropriate temperatures and times to maximize the RUP while avoiding a detrimental decrease in ID [14]. Some nutritional systems, such as the NASEM [12] and BR- CORTE [16], suggest an average ID of RUP of approximately 80%. However, several studies have demonstrated that the ID of RUP can vary substantially depending on the type of feed ingredient and the processing method applied. For instance, different heat treatments applied to soybean meal or canola meal have been shown to significantly alter protein digestibility, either increasing or decreasing it depending on the conditions [3,17,18]. Thus, the results of the literature seemed to confirm that using a single standard ID value does not reflect the characteristics of all feeds, leading to inadequate estimates of MP and dietary imbalance in AA. Therefore, it is necessary to identify the optimal heat-treatment conditions to optimize the RUP content and assess their impact on ID.

Different heating methods will likely have different impacts on feed structure and use different time and energy consumption. Most studies use fire or electrical resistance as a heating source [19,20]. These methods heat a surface that will be in contact with the feed particles.This form of heating occurs from the outside to the inside of the particle through heat transfer by conduction and convection. An example of this technique is using an oven. This method may result in non-homogeneous processing and require longer times to achieve the target RUP content [19]. Infrared radiation, in contrast, transfers heat from the emitter to the feed surface by radiation. However, we currently have heating technologies, such as microwave (**MW**), which use non-ionizing energy and act from the inside out due to rapid oscillation in the electromagnetic field [18]. These technologies are only viable if they reduce RDP or increase RUP, provided the process does not negatively affect the RUP ID. Although previous studies have evaluated microwave or infrared processing individually, information is still limited regarding their direct comparison across different protein-rich feedstuffs while simultaneously evaluating both ruminal degradability and intestinal digestibility of RUP. Therefore, for a more comprehensive assessment of MW as a heat-treatment method for enhancing RUP production, it is essential to compare it with a conventional high-power method, such as infrared (**IR**) radiation.

Thus, we aimed to identify the ideal thermal processing method (MW or IR) to increase the RUP content of the feeds, evaluate the impact on RD and ID, and verify whether the effects are consistent across different protein feeds.

## Materials and methods

This project was approved by the Ethics Committee on the Use of Farm Animals (CEUAP) of the Federal University of Viçosa, protocol 04/2021. This study was carried out at the Animal Nutrition Laboratory, Department of Animal Science, Federal University of Viçosa, between May 2021 and July 2021. No additional permits were required to access the study site because the research was conducted within the University’s facilities.

### Defining treatment conditions

The experiment was conducted in two phases. The first phase aimed to define the heat treatment protocols for soybean meal (**SBM**), using mathematical analyses to estimate the time points at which RUP would stabilize or reach its maximum level. Two thermal treatments were applied: one using a MW device (Panasonic Model, 900 watts, NN-SB438S, China) and the other using an IR lamp (150-watt Philips IR lamp). This phase was based on using SBM as a protein-feed model and allowed us to select optimal time points for each treatment (i.e., IR and MW). SBM was chosen because it is one of the most widely used and well-characterized protein supplements in ruminant diets, with a consistent chemical composition, predictable ruminal degradation characteristics, and low batch-to-batch variation. Its widespread use and extensive characterization have made it a reference ingredient for studies evaluating protein utilization and responses to heat processing, making it an appropriate model for defining thermal processing conditions before applying them to other protein feedstuffs [21–28].

In the second phase, the best results from the first phase, along with the time points for the MW and IR treatments, were applied to six different protein-rich feeds to evaluate the effectiveness of each treatment across other dietary protein sources. We acknowledge that a limitation of this approach is that the preliminary trial was conducted only with soybean meal (SBM), and the resulting time points were used as a reference for the other feedstuffs. Although ingredient-specific pre-tests would have been ideal, using SBM as a model provided a consistent and practical basis for comparing treatments.

### Feed sample collection and preparation

Soybean meal, wheat bran, cottonseed meal, corn gluten feed, whole cottonseed, and whole soybean seed were the protein feeds used in this study (Table 1). To ensure representative sampling, each feed was collected from different regions of Brazil and from different feed mills. Accordingly, four independent samples were collected for each feed by taking a primary sample from 10 distinct increments within the storage bin at the feed mills.

**Table 1:** Chemical characteristics of feeds.

Feed	Dry matter (g/kg)	Crude Protein (g/kg of DM)	RDP¹ (g/kg of CP)	RUP² (g/kg of CP)
Corn gluten feed	953 _± 17.7_	236 _± 6.4_	907 _± 8.3_	93 _± 8.3_
Cottonseed meal	939 _± 18.6_	406 _± 25.6_	850 _± 60.2_	149 _± 60.2_
Soybean meal	944 _± 23.8_	494 _± 14.7_	850 _± 22.5_	149 _± 22.5_
Wheat bran	943 _± 20.6_	162 _± 14.1_	934 _± 3.3_	66 _± 3.3_
Whole cottonseed	954 _± 16.2_	226 _± 16.6_	850 _± 5.6_	149 _± 5.6_
Whole soybean seed	953 _± 27.5_	371 _± 17.0_	634 _± 28.8_	366 _± 28.8_

¹RDP: Rumen Degradable Protein, determined using control data.

²RUP: Rumen Undegradable Protein, determined using control data.

Each primary sample (batch) was then homogenized and split into six subsamples of approximately 415 g each. Considering the six feedstuffs and four independent primary samples per feedstuff, the experiment included 24 independent experimental units (6 feedstuffs × 4 primary samples). Each primary sample was divided into six subsamples corresponding to the three heat treatments (control, MW, and IR), each processed in duplicate. The duplicate heat treatments were considered technical replicates and were not treated as independent experimental units. All samples were properly labeled and stored under appropriate conditions until laboratory analyses were performed.

### First phase – Microwave and infrared radiation test

For the MW test, the four independent primary SBM samples described in section 2.2 (collected from the South, two feed mills in the Southeast, and the Northeast regions of Brazil) were used. Each sample was homogenized and subdivided into test portions, which were then allocated exclusively to MW or IR treatments. Each primary SBM sample was homogenized and split into 100-gram test portions, which were then heat-treated in duplicate. The microwave oven operated at a nominal power of 900 W (9.0 W/g of sample). We used 10-time intervals (0, 1, 5, 10, 20, 30, 38, 45, 60, and 90 minutes), and at each interval, we tested five MW power levels (20%, 40%, 60%, 80%, and 100%). The test portions were evenly spread on the MW glass plate to form a thin layer, and two glasses containing 40 mL of tap water were placed alongside the sample to maintain minimal air humidity, preventing device damage, overheating, and sample combustion. The device was set to operate at the desired power and time, and for MW exposures longer than 5 minutes, the appliance was paused every 5 minutes to replace the water with tap water (room temperature).

At the beginning of each round, MW was run for 10 minutes using 80 mL of tap water in two glasses, without samples, to simulate the conditions required for processing. A pre-test showed that the highest power (100%) should be used, as lower power levels resulted in minimal RUCP gain. Therefore, all samples were processed at 100% power, and the time intervals were adjusted until no further RUCP gain was observed, as detailed in the statistical analysis below.

For the IR test, the same four independent primary SBM samples described in section 2.2 (previously homogenized and subdivided into test portions) were used, with specific portions allocated exclusively to IR treatment. 100 g portions of SBM were separated and subjected to thermal treatment, always in duplicate. The following processing time points were tested: 0, 15, 20, 25, 30, 45, 60, 70, 90, 100, 120, and 150 minutes. A 150-watt Philips IR lamp (1.5 W/g of sample) was fixed inside a cardboard box lined with aluminum foil. The box measured 35 cm high, 30 cm long, and 20 cm wide. The lamp was fixed 20 cm from the box surface (bottom) and suspended by its power supply wire. With these adaptations, we aimed to create a more homogeneous and efficient processing environment. A 100-g test portion was evenly spread on a circular aluminum plate with a 20 cm radius. Additionally, every 5 minutes, the sample was stirred with a stainless-steel spoon, ensuring that a thin, uniform layer of the sample was exposed to IR radiation.

Before processing the sample, we turned on the lamp for 15 minutes with an empty box to create an environment as close as possible to the intended processing period. We used as many time points as necessary to reach a point at which no further gain in RUCP was observed, as described below in the statistical analysis. Subsequently, we conducted 16-hour ruminal incubations Calsamiglia & Stern [17] to determine the RCPD for both IR- and MW-processed SBM samples.

#### In situ incubation procedure.

The *in situ* incubation procedures were performed using four rumen-cannulated Nellore steers, with an average body weight of 544.2 ± 22.7 kg, and 30 ± 0.65 months old. The steers were kept in individual stalls with rubberized floors, feeders, and drinking water troughs, with unrestricted access to water. The steers were fed *ad libitum* a basal diet composed of corn silage and concentrate [forage:concentrate ratio of 80:20 on a dry matter (DM) basis, 16.5% CP]. The concentrate consisted of ground corn, soybean meal, sodium chloride, dicalcium phosphate, urea (2.7% of concentrate DM), ammonium sulfate (0.3% of concentrate DM), and micro-minerals mixture. The steers were fed twice daily (6:00 h and 16:00 h). The animals were adapted to the diet for 14 days before starting the incubations [29].

The ingredients were incubated in rounds due to the large number of samples (144). Thus, each ingredient subjected to thermal treatment (CON, control; MW30, 30 minutes in the microwave; and IR92, 92 minutes in the infrared) was incubated simultaneously in four animals. We performed a 16-hour incubation to determine RCPD [17]. The incubation bags were made of nylon fabric (Sefar Nitex; Sefar, Switzerland), with a 50 μm porosity and dimensions of 8 × 15 cm. Approximately 6 g of air-dried sample was used in each bag, corresponding to about 20 mg DM per cm² of surface area. The bags were arranged in the rumen in reverse order of incubation time so that their removal occurred simultaneously. After removal from the rumen, all bags were washed in running water to remove external residues, then washed in a washing machine for 5 cycles of 1 minute each. Then, the bags were oven-dried (55°C for 72 hours) and weighed. Finally, feedstuffs and incubation residues were analyzed for DM and CP, and a 10-g aliquot of the residues was saved for the *in vitro* digestibility assay [30]. After the trial, the animals were sent back to the university’s permanent herd (retained for future studies).

### Identification of the optimal treatment time for each method to achieve an ideal RCPD

#### Calculations for the first phase.

Initially, two mathematical approaches were applied to determine the optimal time for each treatment: a linear-response plateau model and an exponential model.

To fit the linear-plateau model (i.e., broken-line model), the following equations were used:


$$\begin{array}{c} \left\{ \begin{array}{c} Y_{ij}=\beta_{\text{0}}+\beta_{\text{1}}\times T_{i}+e_{ij},when\;T_{i}<T_{breakpoint}\\ Y_{ij}=\beta_{\text{0}}+\beta_{\text{1}}\times T_{breakpoint}+e_{ij},when\;X_{i}\geq T_{breakpoint} \end{array}\right. \end{array}$$

Where*:* Y_ij_ is the RCPD (% of total CP) measured in the primary sample j submitted to the treatment time i, T_i_ is the treatment time (min), β_0_ and β_1_ are the model parameters, and e_ij_ is the random error.

In this model, T_breakpoint_ represents the time at which there is no further response in Yij and was used as our initial estimate of the critical time point.

A second estimate of optimal treatment time was obtained using the following exponential model:


$$\begin{array}{c} {CPdeg}_{ij}=b\times {exp}^{-k\;\times \;T_{i}}+u+e_{ij} \end{array}$$

Where: CPdeg_ij_ is the RCPD (% of total CP) measured in the primary sample j submitted to the treatment time i, T_i_ is Time (min), b is the parameter that represents the potential variation in the degradable portion of protein, u is the parameter that represents the asymptotic degradable portion of protein, k the is the fractional variation rate of the b fraction, and e_ij_ is the random error.

As the exponential model do not have an inflection point, the moment with maximum marginal drop in CPdeg relative to the average rate (return efficiency peak) was estimated using a geometric approach. First, a linear baseline curve was established by drawing a straight line connecting the first and last data points along the X-axis (time) of the exponential curve. This linear baseline served as a reference representing a constant rate of change across the observed range. Next, the vertical difference between this linear baseline and the estimated exponential curve was calculated along the function domain. These calculated differences were plotted against time. The time point at which the maximum difference was reached was defined as the point of optimized gain in terms of RUCP.

#### Comparison of mathematical approaches for optimal treatment time.

Two heat treatment methods (MW and IR) were evaluated, and the broken-line and exponential models were compared using the Bayesian Information Criterion (BIC), where lower values indicate a better balance between model fit and complexity. The optimal processing times selected for the second phase were 30 min for MW and 92 min for IR, as determined from the mathematical modeling performed in the first phase. These incubation times were subsequently applied to all feed ingredients evaluated in this study.

### Second phase – Application of optimal heating times

In the second phase, the optimal heating times determined in the first phase (30 min for MW and 92 min for IR) were applied to all feedstuffs evaluated in this study (soybean meal, wheat bran, cottonseed meal, corn gluten feed, whole cottonseed, and whole soybean seed). Each primary sample of the six feedstuffs was subjected to one of the two heating methods for the assigned optimal time or kept untreated as the control. The treated samples were then processed and analyzed as described below.

#### Sample processing and chemical analysis.

After the heating treatments, the test portions were ground in a knife mill (Willye type mill, TECNAL, Piracicaba, São Paulo, Brazil) first using a 2-mm screen sieve. This particle size was later used for in situ incubations. Then, approximately 20 g of each test portion was taken and ground again using a 1-mm screen sieve. This particle size was used to analyze DM and N according to the general procedures established by methods G-003/12 and N-001/32, respectively [30] INCT). The CP was calculated as N × 6.25.

### In situ incubation procedure

The *in situ* incubation procedures were performed as previously described in Section 2.3.1.

### In vitro digestibility assay

After quantifying the RCPD content, the residues were used to estimate protein digestion at the intestinal level using three artificial incubators (DaisyII Incubator, Ankom). Each Daisy incubator contains four glass vessels that rotate continuously at 39°C; for this study, three DaisyII units were employed. We used 4 cm × 4.5 cm nylon bags (Sefar Nitex; Sefar, Switzerland) with a porosity of 50 μm. After washing, the nylon bags were oven-dried (55°C for 48 hours) and weighed. With the bags properly labeled, 3 grams of ruminal incubation residues were weighed. Three bags were used for each feed residue.

For the *in vitro* procedure, two enzymatic solutions were used during the incubation. All solutions were prepared in the morning so that incubations could take place in the afternoon. First, a 0.1 M HCl (pH 1.9) solution containing 1 g/L of pepsin (P-7000, Sigma, St. Louis, MO, USA) was prepared. Next, the pancreatin solution was prepared, containing 0.5 M KH_2_PO_4_ (pH 7.75), 50 ppm thymol, and 3 g/L of pancreatin (P-7545, Sigma, St. Louis, MO, USA).

Twenty-five bags were incubated in each Daisy jar. Then, 2 L of the pepsin solution was added to each bottle, and the bottles were kept in constant rotation at 39°C for 1 hour. After this period, the bags were washed in a washing machine for 6 cycles, using washing cycles similar to those used after ruminal incubation, ensuring the washing water was clear at the end of the process. The bags were then incubated in 2 L of the pancreatin solution following the same procedure as for the pepsin solution. These bottles remained in constant rotation for 24 hours at 39°C. After this period, the bags were washed again following the same protocol. Finally, the bags were oven-dried (55°C, forced-air for 72 hours) and weighed. Aliquots of the incubation residues were analyzed for N content as previously described.

### Calculation of protein available for absorption in the small intestine

For the calculation of the proportion of protein available for absorption in the small intestine (Fig 4), we initially entered the CP values analyzed in the laboratory for each of the six feeds. Subsequently, we used the RCPD (%) values obtained from ruminal incubation for the control feed and the treatments MW30 and IR92. Multiplying the CP value by the percentage of RCPD resulted in MCP. Thus, we did not account for any ammonia loss through the rumen wall in those calculations. This value was then corrected to metabolizable protein of microbial origin (**MMP**) by applying the factor of 0.64, which corresponds to the product of two sequential 0.80 factors (0.80 × 0.80): the first represents the proportion of MCP that is true microbial protein, and the second represents the true intestinal digestibility of MCP [12]. After calculating MMP, we obtain the RUCP value for each feed and treatment by subtracting the RCPD value from 100%. We then compared the RUCP values with the *in vitro* digestibility analysis of CP. By multiplying the RCPD and RCPD *in vitro* digestibility values, we obtained the value of digestible RCPD (**RCPDd**). Lastly, the protein available for absorption in the small intestine was calculated by adding the MMP and the RCPDd value.

### Protein secondary structure analysis

To investigate whether protein secondary structure was associated with intestinal digestibility, the amino acid composition of each feed ingredient was obtained from NASEM [12]. Amino acids were classified according to their reported propensity to promote either α-helix or β-sheet secondary structures, based on Yu et al. [31] and Öten et al. [32]. The percentages of amino acids classified as α-helix-promoting and β-sheet-promoting were summed for each feed ingredient, and the proportion of β-sheet-promoting amino acids was expressed as β-sheet/(α-helix + β-sheet). In addition, the combined proportion of glycine and proline was calculated because these amino acids are recognized as helix breakers that increase protein chain flexibility. The estimated structural indices were subsequently correlated with the observed changes in intestinal digestibility following thermal processing (Fig 5).

### Statistical analysis

The results for CP and DM from both the in situ ruminal degradation assay and the *in vitro* intestinal digestibility assay were evaluated using a 6 × 3 factorial design, with six feedstuffs (soybean meal, wheat bran, cottonseed meal, corn gluten feed, whole cottonseed, and whole soybean seed) and three heating treatments (Control, MW30, and IR92). In both analyses, data points were considered outliers when the studentized residual exceeded |2.5|, However, no observations met this criterion, and therefore no data points were excluded from the analyses. The Kenward-Roger procedure was used to adjust the degrees of freedom. Data were analyzed using the GLIMMIX procedure in SAS (University Edition) under a completely randomized design, adopting 0.05 as the critical probability level for type I error.

## Results

### Initial phase results: Maximizing RCPDin Soybean meal with thermal procedures

During phase one, we evaluated the ability of two thermal processing methods (MW and IR) to increase RUP exclusively in SBM (Fig 1). The increase in processing time led to higher SBM RCPD content.

**Fig 1:**
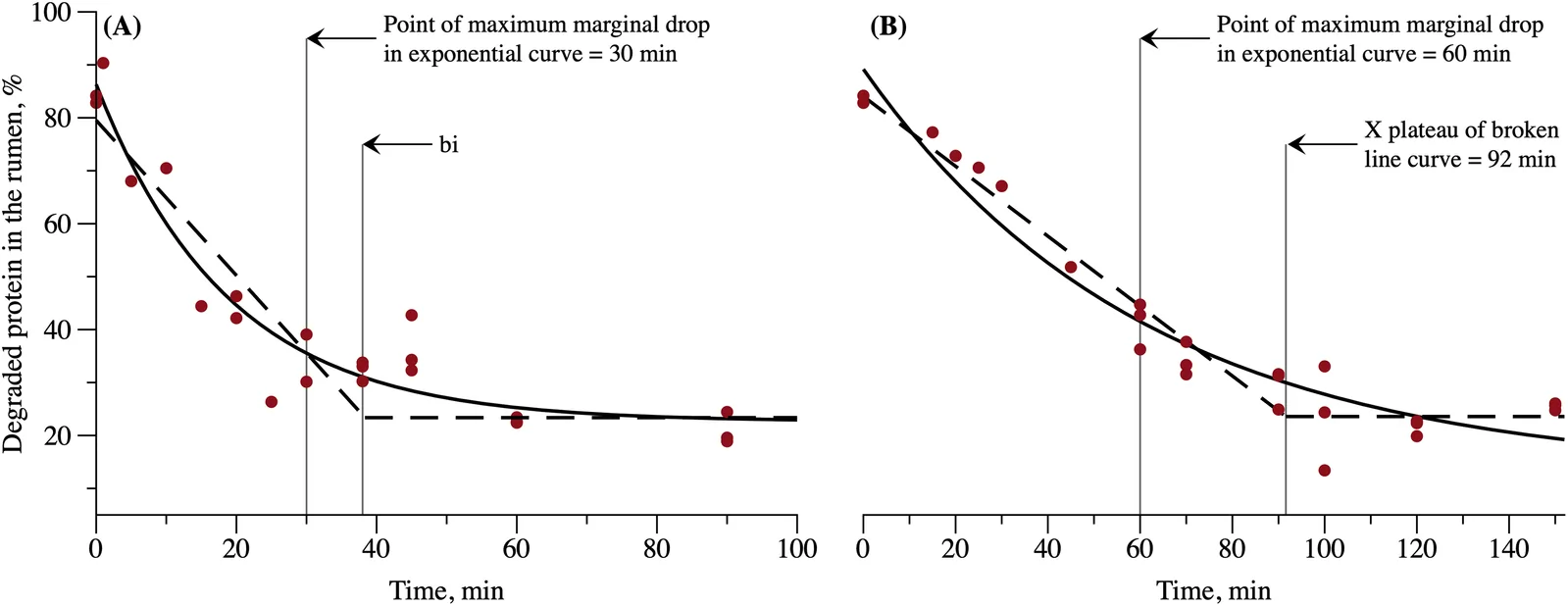
Crude protein degraded in the rumen (%) over time (min) following (A) microwave and (B) infrared thermal processing. Two mathematical approaches were evaluated to estimate the optimal processing time: the exponential model (maximum marginal drop) and the broken-line (linear plateau) model.

Using the linear plateau method (broken-line analysis), we first estimated the time at which RCPD content no longer increases (X plateau) with the MW method. The fitted model was:


$$\begin{array}{c} \left\{ \begin{array}{c} {CPdeg}_{ij}={\text{7}\text{9}.\text{5}\text{1}}_{\pm \text{3}.\text{7}\text{2}}-{\text{1}.\text{4}\text{6}}_{\pm \text{0}.\text{1}\text{6}}\times T_{i},when\;T_{i}<T_{breakpoint}\\ {CPdeg}_{ij}=\text{2}\text{3}.\text{3}\text{8},when\;T_{i}\geq T_{breakpoint}\\ T_{breakpoint}={\text{3}\text{8}.\text{3}\text{3}}_{\pm \text{3}.\text{3}\text{9}} \end{array}\right. \end{array}$$

MSE = 56.40

BIC = 132.55

Where: CPdeg_ij_ is the RCPD(% of total CP), and T_i_ is time (min). These results determined the critical time point to be 38 min (round 38.34 min).

In the second approach, we adjusted the CP ruminal degradation curve according to time using the exponential model:


$$\begin{array}{c} {CPdeg}_{ij}={\text{6}\text{3}.\text{6}\text{6}}_{\pm \text{4}.\text{0}\text{9}}\times {exp}^{-{\text{0}.\text{0}\text{5}\text{3}}_{\pm \text{0}.\text{0}\text{0}\text{9}}\;\times \;T_{i}}+{\text{2}\text{2}.\text{6}\text{3}}_{\pm \text{2}.\text{9}\text{8}} \end{array}$$

MSE = 33.77

BIC = 149.39

Where: CPdeg_ij_ is the RCPD in the rumen (% of total CP), and T_i_ is Time (min). Using this model, the maximum marginal drop, representing the point at which we have the maximum processing efficiency, was determined at 30 min (round of 29.71 min).

The same procedures were then conducted for IR. Firstly, we fitted CP rumen degradability using the linear plateau model:


$$\begin{array}{c} \left\{ \begin{array}{c} {CPdeg}_{ij}={\text{8}\text{4}.\text{0}\text{5}}_{\pm \text{2}.\text{2}\text{1}\text{0}}-{\text{0}.\text{6}\text{6}}_{\pm \text{0}.\text{0}\text{3}\text{8}}\times T_{i},when\;T_{i}<T_{breakpoint}\\ {CPdeg}_{ij}=\text{2}\text{3}.\text{5}\text{8},when\;T_{i}\geq T_{breakpoint}\\ T_{breakpoint}={\text{9}\text{1}.\text{6}\text{1}}_{\pm \text{3}.\text{7}\text{2}\text{1}} \end{array}\right. \end{array}$$

MSE = 18.583

BIC = 173.09

Where: CPdeg_ij_ is CP rumen degradability (% of total CP), and T_i_ is time (min). Based on these results, the first time point was determined as 92 min (round of 91.614 min)

In the second approach, we adjusted the CP rumen degradation curve according to time:


$$\begin{array}{c} {CPdeg}_{ij}={\text{7}\text{6}.\text{3}\text{6}}_{\pm \text{5}.\text{2}\text{5}\text{8}}\times {exp}^{-{\text{0}.\text{0}\text{1}\text{6}}_{\pm \text{0}.\text{0}\text{0}\text{3}}\;\times \;T_{i}}+{\text{1}\text{2}.\text{8}\text{1}}_{\pm \text{5}.\text{4}\text{9}\text{5}} \end{array}$$

MSE = 28.52

BIC = 162.25

Where: CPdeg_ij_ is the RCPD (% of total CP), and T_i_ is Time (min). Using this model, the maximum marginal drop was determined to be at 61 min (round 61.28 min).

For MW, the broken-line (linear plateau) model presented a lower BIC (132.55) than the exponential model (149.39), indicating a better statistical fit. However, the exponential model identified 30 min as the point of maximum processing efficiency, which was selected for the subsequent phase. Conversely, for IR, the exponential model presented a lower BIC (162.25) than the broken-line model (173.09). Nevertheless, the broken-line model identified the plateau at approximately 92 min, which was selected for the subsequent phase. Therefore, MW30 and IR92 were adopted for the second phase.

### Effect of microwave and infrared heating on rumen-degradable protein and RUCP intestinal digestibility

Feed-by-method interaction was significant for both CP rumen degradation and ID (*p* < 0.05). Although the average RCPD of the control ingredients was 82.75%, substantial variation was observed, with values ranging from 63.38% in soybean to 93.35% in wheat bran, which exhibited the highest rumen degradability. When subjected to MW30, all ingredients showed marked reductions in RCPD compared with their respective controls. The largest decrease occurred in wheat bran (93.35% to 39.12%; −54.23 p.p.), followed by soybean meal (85.04% to 31.87%; −53.17 p.p.), cottonseed meal (85.01% to 35.07%; −49.94 p.p.), corn gluten feed (90.70% to 41.46%; −49.24 p.p.), whole soybean (63.38% to 16.94%; −46.44 p.p.), and cottonseed (85.03% to 39.84%; −45.19 p.p.) (p < 0.05). Under IR92, all ingredients also exhibited reductions in RCPD relative to their controls, although the magnitude of these decreases was consistently smaller than under MW30. The greatest reduction under IR92 was observed in cottonseed meal (85.01% to 49.94%; −35.07 p.p.), followed by soybean (63.38% to 32.87%; −30.51 p.p.), soybean meal (85.04% to 59.21%; −25.83 p.p.), cottonseed (85.03% to 59.86%; −25.17 p.p.), corn gluten feed (90.70% to 67.63%; −23.07 p.p.), and wheat bran (93.35% to 77.22%; −16.13 p.p.). All differences were found to be significant (p < 0.05; Fig 2).

**Fig 2:**
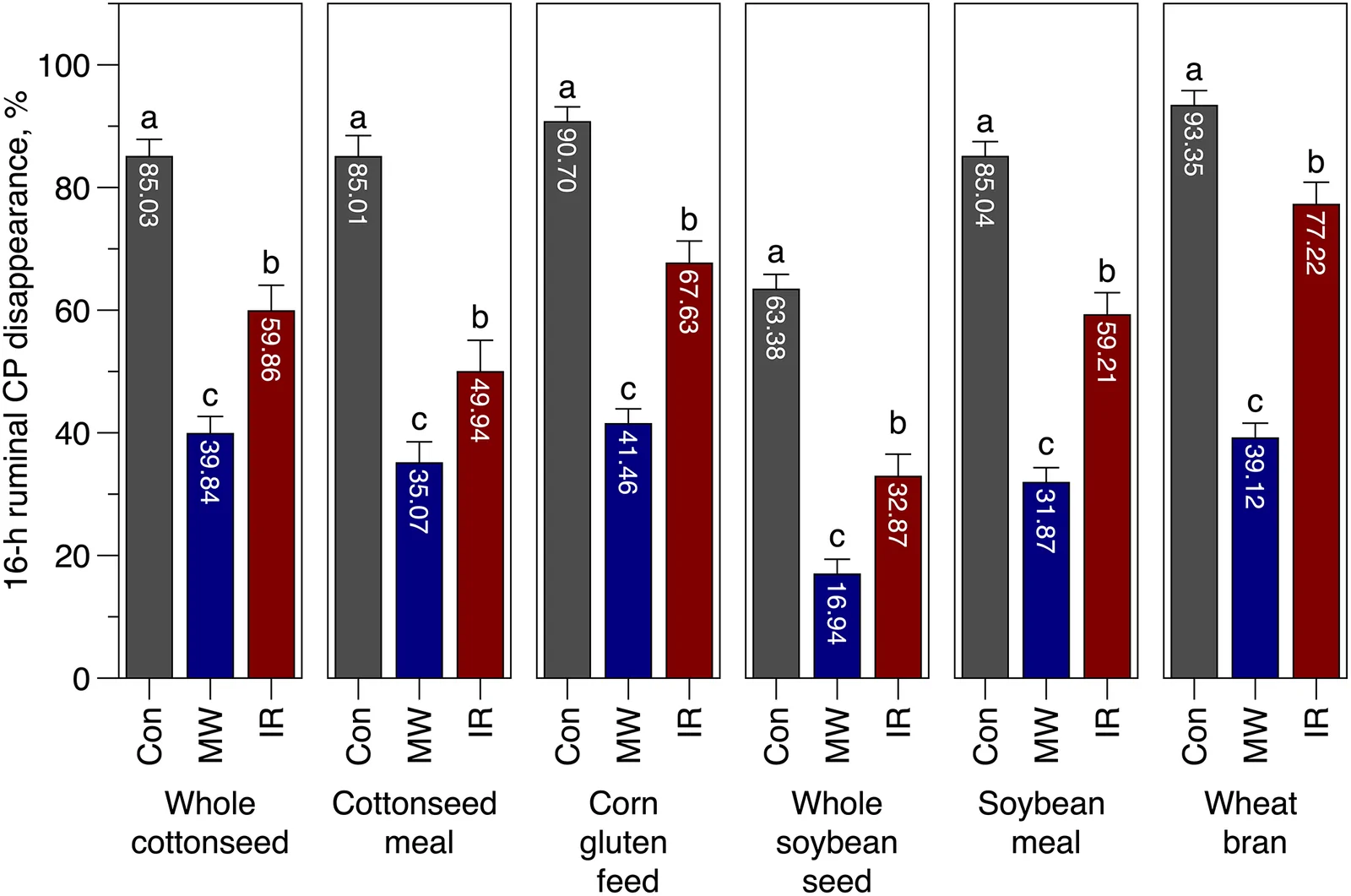
Crude protein ruminal degradability (%) of different feed ingredients. Degradability refers to the percentage of crude protein degraded in the rumen from the original feed. Feeds were subjected to thermal processing by microwave for 30 minutes (MW30) and infrared radiation for 92 minutes (IR92). Bars represent the mean ± SEM (n = 4). Different lowercase letters indicate differences among treatments within each feed ingredient (P < 0.05).

The in vitro intestinal digestibility of RUCP responded differently across ingredients and thermal treatments. In whole cottonseed, both MW30 and IR92 increased digestibility relative to the control (64.13%), reaching 79.31% and 79.24%, respectively (+15.18 and +15.11 p.p.). These two treatments did not differ (p > 0.05). In cottonseed meal, digestibility remained similar across treatments (72.19%, 68.10%, and 63.55% for the control, MW30, and IR92, respectively; p > 0.05). In corn gluten feed, MW30 significantly reduced digestibility (55.80% to 32.50%; −23.30 p.p.; p < 0.05), whereas IR92 maintained values comparable to the control (55.76%; p > 0.05). In soybean, MW30 also caused a pronounced reduction (96.74% to 59.83%; −36.91 p.p.; p < 0.05), while IR92 preserved high digestibility, remaining similar to the control (95.54%; p > 0.05). Digestibility in soybean meal was consistently high and did not differ among treatments (98.45%, 90.50%, and 98.57%; p > 0.05). In wheat bran, MW30 resulted in only a slight, non-significant increase (32.33% to 36.17%; + 3.84 p.p.; p > 0.05), whereas IR92 substantially improved digestibility (59.98%; + 27.65 p.p.; p < 0.05). Overall, IR92 tended to enhance RUP intestinal digestibility, especially in ingredients with low initial values such as wheat bran and cottonseed. In contrast, MW30 reduced digestibility in heat-sensitive ingredients, particularly soybean and corn gluten feed (Fig 3).

**Fig 3:**
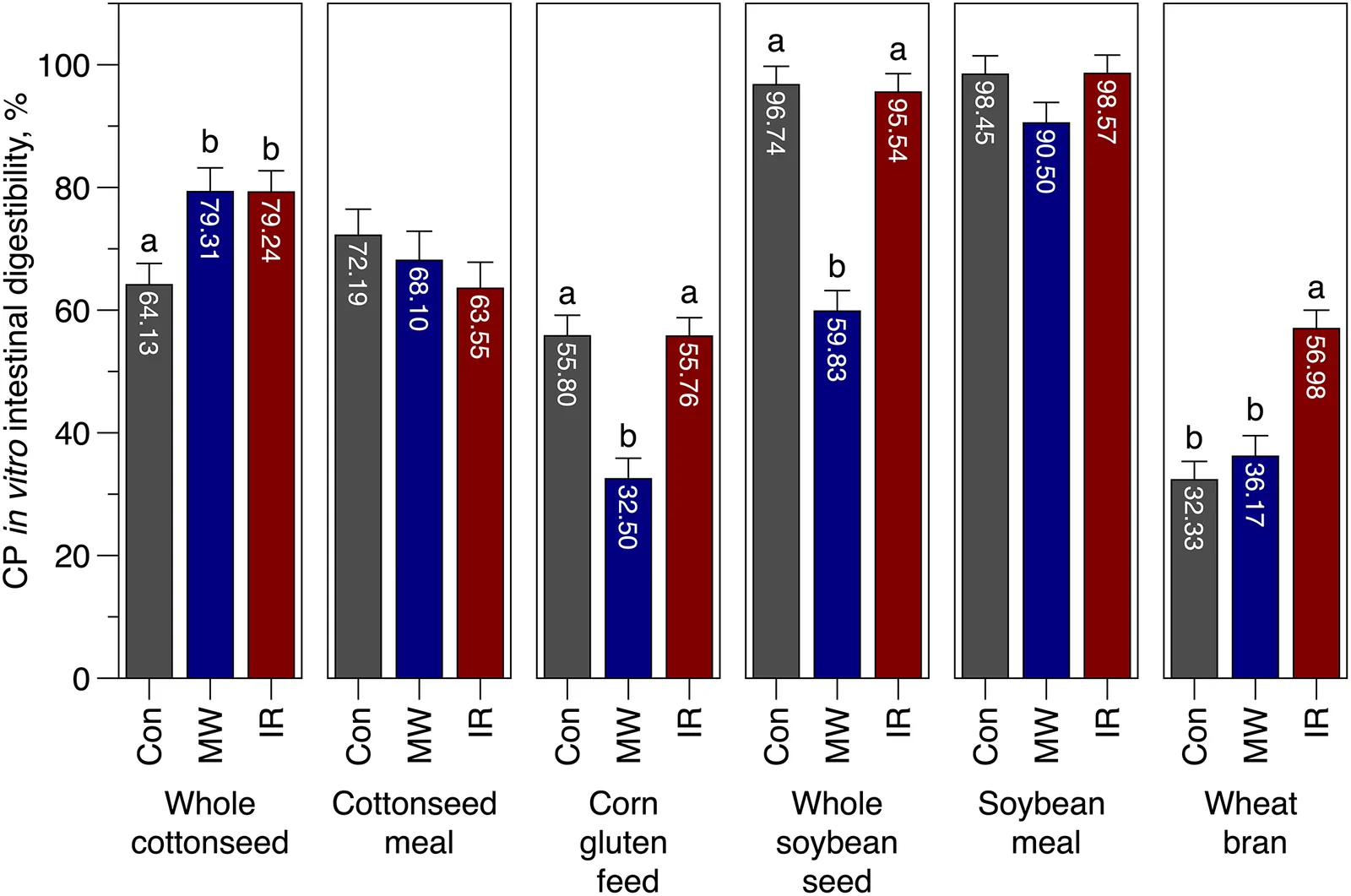
Crude protein in vitro digestibility (%) represents the intestinal digestibility of the 16-h ruminal undegraded crude protein (RUCP) from the incubated residual material. Feeds were subjected to thermal processing by microwave for 30 minutes (MW30) and infrared radiation for 92 minutes (IR92). Bars represent the mean ± SEM (n = 4). Different lowercase letters indicate differences among treatments within each feed ingredient (P < 0.05).

### Effect of processing time and method on proportion of protein available for absorption in the small intestine

Considering that this study has a methodological focus, the main objective when analyzing the increase in intestinal digestibility of rumen undegraded protein (**ID**_**RUP**_) for the tested feedstuffs is to identify the ideal type and duration of thermal processing for each ingredient and to determine whether these treatments promote an increase in the protein available for absorption in the small intestine compared to the control.

Whole soybean seed showed the greatest improvement under infrared treatment, increasing absorbable protein from 75.5% in the control to 85% (Fig 4). Soybean meal also responded favorably to IR, reaching 78%, compared with 69% in the control. Wheat bran and corn gluten feed showed limited sensitivity to IR, maintaining values close to their respective controls (61.5% and 61%). In contrast, microwave processing produced the strongest responses in cotton-based ingredients. Whole cottonseed increased from 64% to 73.5% with MW, while soybean meal reached the highest MW-treated value among all feeds (81%). Cottonseed meal remained relatively stable across treatments, ranging from 64% to 66%. However, MW reduced absorbable protein in wheat bran and corn gluten feed, yielding the lowest values observed for these ingredients (49% and 46%, respectively).

**Fig 4:**
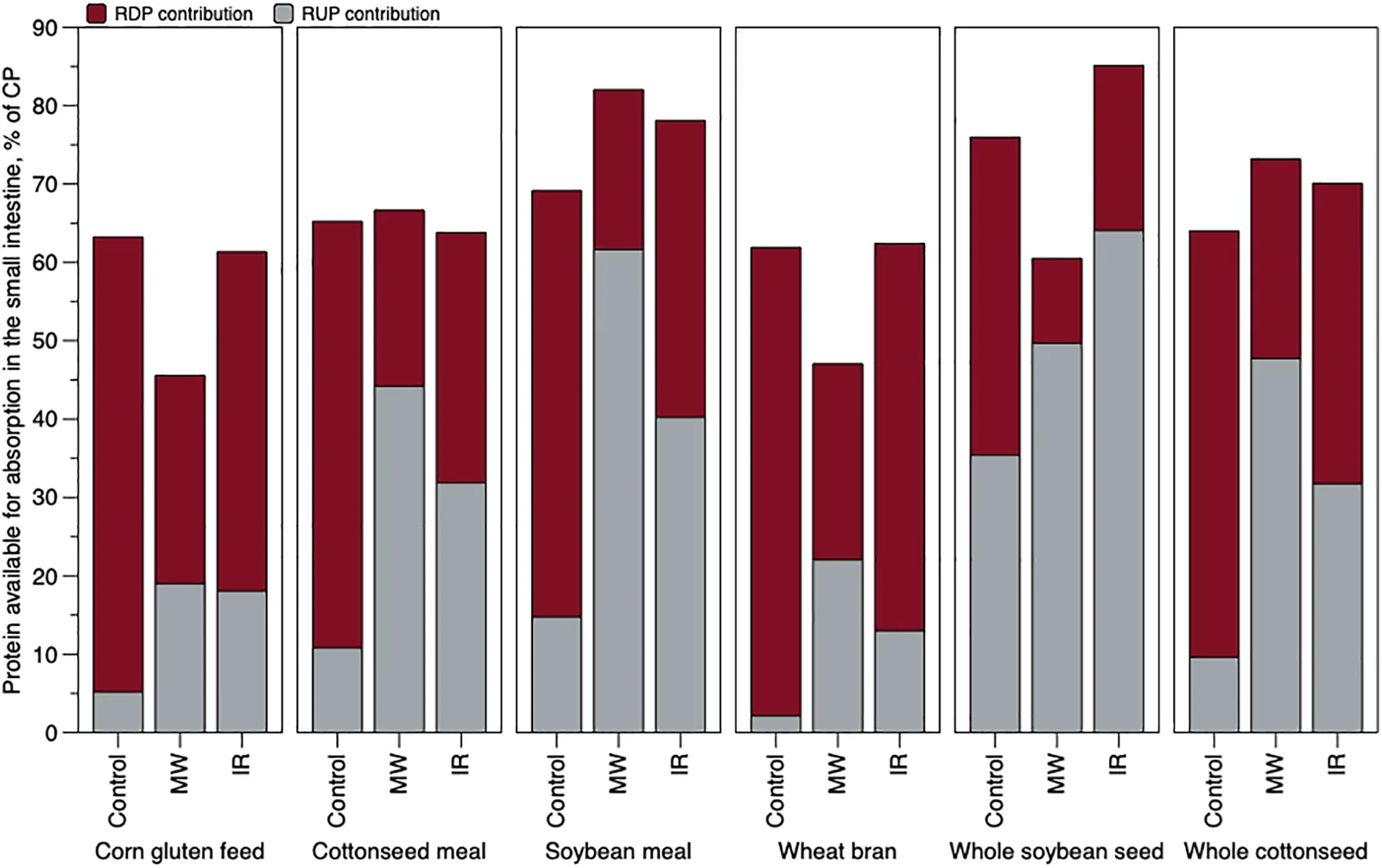
Proportion of protein available for absorption in the small intestine for each feed, method, and thermal processing time. The fraction labeled as “RCPD contribution” represents microbial protein synthesized from RCPD and corrected for true microbial protein and intestinal digestibility (MCP × 0.64) [12], and “RUCP contribution” represents 16-h ruminal undegraded crude protein digestible in the small intestine. This fraction does not represent undegraded dietary protein, but the portion of microbial protein ultimately available for absorption.

## Discussion

The different optimal processing times observed for MW and IR heating reflect the interaction between feed protein structure and the distinct mechanisms of energy transfer associated with each method. The relative proportions of secondary structures, such as α-helix and β-sheet, directly influence ruminal degradability and intestinal digestibility because these conformations differ in thermal stability and enzymatic susceptibility [31,33,34]. Heating can also trigger Maillard reactions, forming insoluble nitrogenous compounds that reduce post-ruminal digestibility [35–38]. Thus, how each technology distributes heat within the feed matrix determines both the rate and magnitude of these structural alterations.

MW processing promotes volumetric energy absorption via dipole rotation and ionic conduction, resulting in rapid internal heating and localized “hot spots” [39]. Deng et al. [40] demonstrated that this heating pattern induces intense structural changes during the initial minutes, followed by stabilization, a behavior that explains the superior fit of the exponential model for MW30. In contrast, IR radiation first heats the surface, with the energy conducted gradually inward [41]. This process generates an initial linear reduction in degradability followed by a plateau, which is accurately described by the broken-line model [42]. Consistent with these thermodynamic principles, MW required a shorter optimal time (30 min), whereas IR required a longer exposure time (92 min). Excessive MW exposure intensifies advanced Maillard reactions and can impair intestinal digestibility [43], whereas IR tends to better preserve post-ruminal digestion [44,45].

The magnitude of the thermal response varied markedly among ingredients, reflecting differences in structural organization, chemical composition, and protein matrices. Ingredients rich in insoluble fiber and reactive structural carbohydrates, such as wheat bran and cotton by-products, tend to form protein–carbohydrate crosslinks under heating, reducing protein solubility and increasing ruminal escape [46]. Accordingly, wheat bran exhibited the greatest RDP reduction under MW30, consistent with its high arabinoxylan content and elevated susceptibility to Maillard reactions [47].

Concentrated protein sources also showed strong thermal sensitivity, but through distinct mechanisms [48]. In soybeans, the globular proteins glycinin (11S) and β-conglycinin (7S) are highly sensitive to overheating, denaturing rapidly and losing solubility under MW [34]. This explains the pronounced decline in RCPD under MW30, whereas IR92 produced more moderate reductions, consistent with its slower, surface-limited heating. Corn gluten feed, however, exhibited a different response due to its high zein content, a hydrophobic and thermally stable prolamin [49]. Zeins resist gradual heating but readily form insoluble aggregates under rapid, high-intensity heating such as MW [50], resulting in the greater RCPD reduction observed with MW.

Differences between whole cottonseed and cottonseed meal further highlight the role of matrix microstructure. Whole cottonseed retains a lignified hull and higher lipid content that act as barriers to heat diffusion, slowing protein denaturation. Cottonseed meal, with a more exposed and homogeneous protein matrix, is more susceptible to thermal modification [51].

Intestinal digestibility of RUCP also responded differently across treatments. MW30 markedly reduced digestibility in soybean and corn gluten feed, consistent with hotspot formation, irreversible denaturation, and intensified Maillard reactions [40]. In corn gluten feed, this reduction was amplified by zein aggregation [50]. In soybeans, digestibility declined due to thermal aggregation of 11S and 7S globulins, forming low-solubility protein complexes that resist enzymatic hydrolysis [52]. Conversely, IR92 preserved or enhanced digestibility in soybean meal, whole cottonseed, and wheat bran, reflecting its gradual, surface-oriented heating that favors reversible denaturation and limits insoluble aggregate formation [39,42]. These results indicate that reductions in ruminal CP disappearance are desirable only when intestinal digestibility is preserved. Although MW30 effectively increased ruminal protein escape in soybean meal and corn gluten feed, the concurrent reduction in intestinal digestibility suggests that part of the protected protein became less available for post-ruminal digestion. Therefore, the nutritional value of thermal processing should be assessed by considering both ruminal protection and intestinal digestibility rather than reductions in ruminal degradation alone.

Part of these responses may also be interpreted through protein conformational composition. Amino acids that favor β-sheet formation (e.g., phenylalanine, tyrosine, valine, isoleucine) stabilize rigid, less hydrated structures, whereas glycine and proline act as “helix breakers,” increasing chain flexibility and favoring digestibility [31,32]. Based on AA profiles [12], the estimated β-sheet:α-helix ratios correlated negatively with intestinal digestibility, while glycine + proline content correlated positively (Fig 5). Wheat bran and whole cottonseed, with lower β-sheet proportions and higher glycine + proline, showed the greatest digestibility improvements under IR92, whereas corn gluten feed, rich in β-sheet–dominant prolamins, exhibited consistent reductions across both methods [15,53,54].

**Fig 5:**
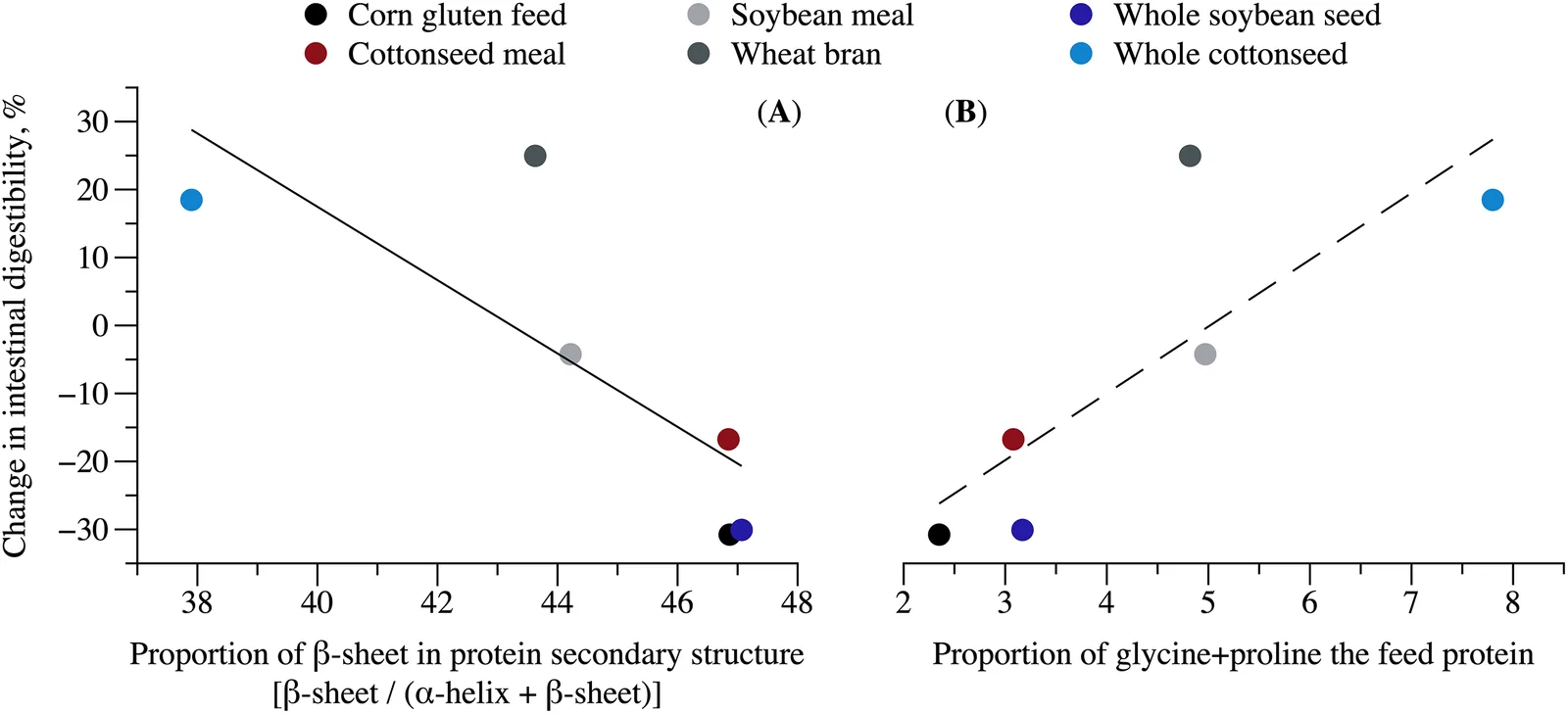
Change in intestinal digestibility (%) – The relationship between the proportion of protein secondary structures and the proportion of two amino acids (glycine and proline) with the intestinal digestibility of feeds.

These findings reinforce that reducing ruminal CP disappearance does not necessarily increase intestinal protein supply. Excessive heating can compromise post-ruminal digestibility [44,45]. Moreover, part of metabolizable protein derives from microbial protein (MCP), which has a true digestibility of ~64% [12]. Although useful for standardized comparisons, this estimate does not account for potential changes in microbial efficiency driven by alterations in nitrogen availability and by thermal processing-induced synchronization [55]. Therefore, defining optimal processing conditions requires balancing ruminal protection with preservation of intestinal digestibility, taking into account the ingredient- and method-specific responses observed in this study.

## Conclusion

The thermal processing methods evaluated produced ingredient-specific responses driven by structural characteristics. Microwave heating (MW30) was most effective for whole cottonseed, which showed the greatest increase in absorbable protein, whereas it reduced intestinal digestibility in heat-sensitive ingredients such as soybean and corn gluten feed. Infrared radiation (IR92) performed best for thermally sensitive protein sources, particularly whole soybean, which exhibited the greatest improvement under this method. Overall, MW30 is better suited for fibrous and structurally stable ingredients, while IR92 is preferable for ingredients rich in globular, heat-sensitive proteins.

### Applications

These results can help guide the selection of appropriate thermal processing methods according to feed ingredient characteristics. However, additional studies under commercial-scale processing conditions are needed before these findings can be directly applied by the feed industry to optimize the proportion of protein available for absorption in the small intestine, improving feed efficiency and sustainability in ruminant production systems. In addition, the ingredient-specific responses observed in this study may also have implications for thermally processed companion animal and avian feeds, where processing conditions and feed matrix can influence nutrient availability and product quality [56,57]. Future studies should also evaluate the scalability of microwave and infrared processing, as well as microwave and infrared pretreatments before extrusion in these feed systems.

## Supporting information

S1 File Inclusivity in Global Research Questionnaire for the present study. (DOCX)

S1 Data Dataset underlying the results presented in this study. (XLSX)

## Abbreviations

MW: microwave
MW30: 30 minutes in the microwave
MW38: 38 minutes in the microwave
IR: infrared
IR60: 60 minutes in the infrared
IR92: 92 minutes in the infrared
MCP: Microbial crude protein
MP: metabolizable protein
AA: amino acids
ID: intestinal digestibility
CP: crude protein
ID_RUP_: Intestinal Digestibility of RUP
N: nitrogen
RD: ruminal degradability
RUCP: 16-h ruminal undegraded crude protein
RUP: rumen undegradable protein
RCPD: 16-h ruminal crude protein disappearance
RDP: rumen degradable protein
MMP: metabolizable protein of microbial origin
RUPd: digestible rumen undegradable protein
